# Exosomes Carry microRNAs into Neighboring Cells to Promote Diffusive Infection of Newcastle Disease Virus

**DOI:** 10.3390/v11060527

**Published:** 2019-06-06

**Authors:** Changluan Zhou, Lei Tan, Yingjie Sun, Xusheng Qiu, Chunchun Meng, Ying Liao, Cuiping Song, Weiwei Liu, Venugopal Nair, Chan Ding

**Affiliations:** 1 Shanghai Veterinary Research Institute (SHVRI), Chinese Academy of Agricultural Sciences (CAAS), Shanghai 200241, China; changluanzhou@163.com (C.Z.); sunyingjie@shvri.ac.cn (Y.S.); xsqiu1981@shvri.ac.cn (X.Q.); mengcc@shvri.ac.cn (C.M.); liaoying@shvri.ac.cn (Y.L.); scp@shvri.ac.cn (C.S.); liuweiwei@shvri.ac.cn (W.L.); 2 The Pirbright Institute, Ash Road, Pirbright, Woking, Surrey Gu24 ONF, UK; venugopal.nair@pirbright.ac.uk; 3 Jiangsu Co-innovation Center for Prevention and Control of Important Animal Infectious Diseases and Zoonoses, Yangzhou 225009, China

**Keywords:** NDV, oncolytic, exosomes, microRNA (miRNA), IFN-β

## Abstract

Newcastle disease virus (NDV), an avian paramyxovirus, was shown to prefer to replicate in tumor cells instead of normal cells; however, this mechanism has not been fully elucidated. Exosomes play a crucial role in intercellular communication due to the bioactive substances they carry. Several studies have shown that exosomes are involved in virus infections. However, the effect that exosomes have on NDV-infected tumor cells is not known. In this study, we focus on the role of exosomes secreted by NDV-infected HeLa cells in promoting NDV replication. Three miRNA candidates (miR-1273f, miR-1184, and miR-198) embraced by exosomes were associated with enhancing NDV-induced cytopathic effects on HeLa cells. Furthermore, luciferase assays, RT-qPCR, and enzyme-linked immunosorbent assay (ELISA) all demonstrated that these miRNAs could suppress interferon (IFN)-β gene expression. Enhanced NDV replication in HeLa cells was identified by Western blot and plaque assays. Based on these results, we speculate that NDV employed exosomes entry into neighboring cells, which carry miRNAs, resulting in inhibition of the IFN pathway and promotion of viral infection. To our knowledge, this is the first report on the involvement of NDV-employed exosomes in tumor cells, and as such, it provides new insights into the development of anti-tumor therapies.

## 1. Introduction

Newcastle disease virus (NDV), a member of the *Avulavirus* genus of the family *Paramyxoviridae*, is a single-stranded, non-segmented, negative-sense RNA virus. Although NDV is an avian virus, its oncolytic properties have been widely reported [1]. Due to its ability to destroy selectively mammalian tumor cells without any effect on normal cells [2], NDV has shown its potential for cancer treatment [3]. In fact, NDV prefers replication in tumor cells, ultimately killing the tumor cell [4]. NDV not only replicates in tumor cells, which results in the selective elimination of these cells, but it also induces the activation of an anti-tumor response in immune cells. Thus, the tumor selectivity of NDV was associated with limited interferon responses in human cancer cells.

Exosomes are approximately 100–150 nm vesicles with a lipid bilayer. Almost all types of cells and tissues can secrete exosomes. These vesicles can participate in cellular interaction because they can carry various functional RNAs, proteins, and lipids, based on the current state of their parent cells. One of the main functions of exosomes is the involvement in viral pathogenesis and immune responses. The effect of exosomes on viral infection can be either positive or negative, depending on the virus species and targeted cells. For example, exosomes isolated from human immunodeficiency virus (HIV) infected cells contained negative regulatory factors [5], transactivation response elements [6], viral microRNAs (vmiR-88, vmiR-99, and vmiR-TAR) [7], CCR5 [8], and CXCR4 [9]—all of which facilitate HIV-1 invasion. Hepatitis C virus (HCV) utilizes exosomes to transfer its genomic RNA [10], antisense RNA [11], and proteins [12,13], which aid the virus in establishing a productive infection. Exosomes can also confer membranes on non-enveloped viruses, such as hepatitis A virus (HAV) [14], which allows the virus to escape host immune recognition. Exosomes deriving from liver nonparenchymal cells can transmit interferon (IFN)-α induced antiviral activity to hepatitis B virus (HBV) replication hepatocytes [15]. Thus, exosomes are involved in the life cycle of a number of viruses. Moreover, exosomes are also demonstrated to take part in the process of cancer development and show great potential in cancer treatment [16,17].

miRNAs are small RNA regulatory molecules approximately 22 nucleotides in length that are involved in a variety of biological processes, such as cell proliferation, differentiation, tumorigenesis, and immune defense [18,19]. They are usually integrated into complexes with proteins, such as Dicer and Argonaute, to form functional RNA-induced silencing complexes [20]. In animals, miRNA-mediated gene regulation occurs through targeting partially complementary sequences in the 3′ untranslated regions (3′ UTR) of gene mRNAs in order to dampen their translation. RNA sequencing analysis has shown that microRNAs are the most abundant in exosomal RNA species. Exosomal RNAs have been closely related to a multitude of exosome-related biological functions. Recent evidence has demonstrated that exosomal miRNA can not only be secreted by cancer cells, but it can also be taken up by these cells [21]. Importantly, it was proven in a select way that miRNAs were loaded into these exosomes. This leads us to hypothesize that exosomes may transfer miRNAs into the intercellular space in order to assist viruses to escape immune responses in adjacent cells.

IFN-β, one kind of interferons (IFNs), is an inaugural event that occurs in host innate immune responses during viral infection. Only a minimal change in IFN-β expression is needed to activate crucial antiviral signaling cascades [22]. However, there were few studies about the relationship between IFN-β and microRNAs during NDV infection and it remains to be interpreted. 

Exosomes were reported to be involved in the replication of a variety of viruses; however, there are no reports regarding the role of exosomes in NDV infection. In this current study, HeLa cells incubated with purified exosomes showed more serious cytopathic effects during NDV infection. In order to further study the role of exosomes in NDV replication, miR-1273f, miR-1184, and miR-198 in exosomes were found to suppress interferon (IFN)-β mRNA and protein expression. Furthermore, these three candidate microRNAs also promoted NDV replication. We also found that miRNAs via exosomes were released into the extracellular space, which increased the susceptibility of neighboring cells to NDV. Our study describes the role of exosomes carrying host miRNAs in enhancing NDV diffusive infection by regulating the IFN signaling pathway of the host.

## 2. Material and Method

### 2.1. Cells and Virus

HeLa cells, 293T and DF-1 cells were both obtained from the American Type Culture Collection (ATCC, Manassas, VA, USA). These cells were maintained in Dulbecco’s modified Eagle’s medium (DMEM) supplemented with 10% fetal bovine serum (FBS, Thermo Fisher, Carlsbad, CA, USA), 100 IU/mL penicillin, and 100 mg/mL streptomycin at 37 °C with 5% CO_2_. The NDV strain Herts/33 was isolated and identified at the China Institute of Veterinary Drug Control (Beijing, China) [23]. 

### 2.2. Cultured Medium Collection and Exosome Isolation

HeLa cells were infected with NDV at multiplicity of infection (MOI) of 1 and cultured for 24 h. Exosomes were isolated from the supernatants of these cells by differential centrifugation methods according to Thery et al. [24] with slight modifications. Briefly, the first centrifugation step was 300× *g* for 10 min, the second centrifugation step was 2000× *g* for 10 min, and the third centrifugation step was 10,000× *g* for 0.5 h. Ultracentrifugation (L-100XP, Beckman, Indianapolis, IN, USA) was carried out on the supernatants at 140,000 × *g* for 1.5 h. Then the supernatants were discarded, and phosphate-buffered saline (PBS) was added to resuspend the precipitates. The samples were then centrifuged again at 140,000× *g* for 1.5 h. The exosome sample was resuspended in 200 μL PBS and stored at −80 °C.

### 2.3. Exosome Purification

The exosome samples were first analyzed with hemadsorption tests. Then packed chicken red blood cells prepared by our group were added into the sample to adhere the remaining NDV. Finally, the sample was tested by hemadsorption (HA) tests again in order to ensure no virus residue. The step of adhering NDV with red blood cells was repeated for several times until the HA result was negative.

### 2.4. Transmission Electron Microscopy (TEM)

A 15-μL aliquot of the exosome preparation was rinsed with 0.1 M PBS for 2–3 min and applied to the grids. The grids were dried at room temperature for 1–2 min, and then any excess liquid in the grids was removed. The grids were then stained with 2% phosphotungstic acid (PTA) for 60 s, followed by drying for 5 min. The grids were examined at an acceleration voltage of 80 kV using a TEM (Tecnai G2 Spirit BIOTWIN, FEI, Eindhoven, Netherlands).

### 2.5. Nanoparticle Tracking Analysis (NTA)

The NTA experiment was carried out using Zetaview PMX 120 (Particle Metrix, Meerbusch, Germany). Briefly, a 2 mL sample was diluted with 1 × PBS and added into a new cell. The instrument measured 11 different positions of the cell, and the outliers of those measurements were removed. The average and median sizes and the concentration of the particles were calculated based on the data of the optimized positions. In this study, the optimal measurement condition settings were: temperature at 25 °C, sensitivity at 70 °C, shutter speed at 70 s, and conductivity at 15,000 μs/cm.

### 2.6. Western Blot Analysis

The protein samples were isolated with sodium dodecyl sulfate-polyacrylamide gel electrophoresis (SDS-PAGE) and electrotransferred to a nitrocellulose (NC) membrane (Bio-Rad, Hercules, CA, USA). The membrane was then incubated in Tris-buffered saline with Tween 20 (TBST) and 5% skimmed milk for 2 h at room temperature. The membranes were washed three times with TBST and then incubated with the primary antibody overnight at 4 °C. The membrane was cultured with the diluted (1:8000) secondary antibody (anti-mouse and anti-goat) for 1 h at room temperature. Next, the membrane was washed with TBST three times and each time lasted 10 min to remove the superfluous antibodies. Immunoreactive bands were detected using an enhanced electrochemiluminescence (ECL) system (Shanghai Share Biotechnology Co. Ltd., Shanghai, China) and the integrated density of these bands were analyzed using the ImageJ software 1.8.0. Primary antibodies against CD63, CD9 and CD81 (Abcam, Cambridge, UK) and nucleocapsid protein (NP, prepared by our group) were used.

### 2.7. Plaque Assay for NDV Titers

The collected virus was added to Dulbecco’s modified Eagle’s medium (DMEM) in the correct proportion to prepare the serial viral dilutions. A total of 300 μL of each dilution were added into 12-well plates to infect DF-1 cells and were incubated for 1 h. These cells were overlaid with 1.75% methyl cellulose and DMEM containing 2% FBS. At 72 h post-infection, the cells were fixed overnight in 4% phosphate-buffered saline (PBS)-buffered formaldehyde. The next day the cells were stained with 0.5% crystal violet for 2 h and washed with ddH_2_O three times. Finally, plaques formed by the virus were counted, and the diameters of these plaques were analyzed with the Image-Pro Plus 6.0 software (Media Cybernetics, Rockville, MD, USA).

### 2.8. MicroRNA Array

Total RNA was isolated from HeLa cell-derived exosomes by TRIzol reagent (Thermo Fisher, Carlsbad, CA, USA) and purified by a RNeasy mini kit (QIAGEN, Dusseldorf, Germany). The RNA quality and integrity were analyzed with a NanoDrop-1000 spectrophotometer (Thermo Fisher, Carlsbad, CA, USA) and gel electrophoresis, respectively. The miRNA with superior quality was labeled with a miRCURY™ Hy3™/Hy5™ Power labeling kit (Exiqon, Vedbaek, Denmark) as previously described [18]. Following miRNA labeling, the Hy3™-labeled samples were hybridized on a miRCURY LNA™ microRNA Array (v.18.0) (Exiqon, Vedbaek, Denmark). Briefly, a total of 50 μL mixture (25 μL hybridization buffer and 25 μL Hy3™-labeled samples) were first denatured at 95 °C in the dark for 2 min and then placed in an ice bath for 2 min. Next, the hybridization process between the mixture and the microarray lasted for 20 h in a 12-Bay Hybridization System (Hybridization System-Nimblegen Systems, Inc., Madison, WI, USA) at 56 °C. Finally, the hybridized slides were washed with a Wash Buffer Kit (Exiqon, Vedbaek, Denmark) several times and scanned in an Axon GenePix 4000B microarray scanner (Axon Instruments, Foster City, CA, USA).

### 2.9. MicroRNA Target Predictions

Sequences of mature human miRNA candidates were obtained from the miRNA miRbase Registry (http://www.mirbase.org/). The prediction algorithms RNAhybrid [25], miRDB [26], and TargetScan [27] were used to predict miRNAs targeted with human IFN-β 3′ UTR.

### 2.10. Transient Transfection of miRNA Mimics

MiRNA mimics were purchased from Shanghai GenePharma Co. (China). MiRNA mimic powder was dissolved in diethyl pyrocarbonate (DEPC)-treated water and transfected using Lipofectamine 2000 transfection reagent (Thermo Fisher, Carlsbad, CA, USA) into HeLa cells at 70–80% confluences. For each transfection, as previously described [28], 90 pmol of miRNA mimics and 2.5 μL Lipofectamine 2000 were mixed with 250 μL Opti-MEM reduced-serum medium (Thermo Fisher, Carlsbad, CA, USA), respectively. After 5 min, the two mixtures were mixed for 0.5 h at room temperature, and then incubated in HeLa cells for 12 h. Finally, 1.5 mL DMEM supplemented with 2% FBS was added to the wells of a 6-well plate. MicroRNA mimics negative control (NC, Shanghai GenePharma Co., Shanghai, China) is a negative control.

### 2.11. Reverse Transcription Reaction and Quantitative Real-Time PCR (RT-qPCR)

Total RNA from HeLa cells or supernatant NDV RNA were isolated using TRIzol (Thermo Fisher, Carlsbad, CA, USA) and reverse transcribed with Moloney murine leukemia virus (M-MLV) reverse transcriptase (Promega, Madison, USA). The oligo dT (18T) was a primer for reverse transcribing host gene mRNA. Quantitative PCR (qPCR) was carried out using SYBR Green qPCR Mix (Dongsheng, Guangzhou, China) in a 20-μL reaction system with qPCR primers (as shown in Table 1). β-Actin mRNA was used as an internal control to detect host mRNA expression, and the detection of supernatant NDV RNA was performed using the specific primers, as previously described [29].

For the detection of miRNAs, total RNA was reverse transcribed with the RT primers (as shown in Table 2). qPCR was also performed using SYBR Green qPCR Mix (Dongsheng, Guangzhou, China) with the specific qPCR primers, which were designed with the stem-loop method, while U6 small nuclear RNA was used as an internal control to detect miRNA expression (as shown in Table 2).

### 2.12. IFN-β Enzyme-Linked Immunosorbent Assay (ELISA)

HeLa cells were transfected with miRNA mimics using Lipofectamine 2000. After 12 h, the cells were washed with PBS three times and incubated with 50 μg/mL polyinosinic-polycytidylic acid (poly (I:C)) (Thermo Fisher, Carlsbad, CA, USA) or infected with 1 MOI of NDV for 24 h. The IFN-β protein expression was measured in these cultured supernatants by ELISA (R&D Systems, Minneapolis, MN, USA) according to the manufacturer’s protocol. The ELISA results were obtained using a microplate reader, ELX800 (Bio-Tek, Winooski, VT, USA).

### 2.13. Luciferase Assays

The 3′ UTR of human IFN-β mRNA was generated by PCR and cloned between the XhoI and HindIII restriction sites of the luciferase pGL3 basic vector (Promega, Madison, WI, USA). The forward primer sequence was 5′-GCATCGCTCGAGAGATCTCCTAGCCTGTGCC-3′, and the reverse primer sequence was 5′-CGATGCAAGCTTTGACTTTTGCACCAAAAATAATTTATTTTC-3′. 293T cells were co-transfected with pGL3 basic (containing or not containing the IFN-β 3′ UTR sequences) and miRNA mimics. After transfecting for 24 h, the cell lysates were prepared for the measurement of luciferase levels using a TransDetect Double-Luciferase Reporter Assay Kit (TransGen Biotech, Beijing, China).

### 2.14. Preparation of Liposomes Carrying miRNAs

The mixture (4 mg granulesten, 4 mg DOTAP, and 2 mg cholesterol) was dissolved in chloroform and then distilled through reduced pressure to remove organic solvents. For complete dehydration, the mixture was placed into a vacuum dryer overnight. A liposomal suspension was obtained from the hydration of the film in a 50 °C water bath for 15 min following the addition of 3 mL pre-heated water at the same temperature. The suspension was subjected to ultrasound using a JX-IID ultrasonic cell pulverizer (Jingxin, Shanghai, China) (80 W, 10 s, 10 s, 5 times) and filtered through a 0.22-μm polycarbonate membrane to obtain vacant cationic liposomes. To prepare liposomes carrying miRNAs, miRNA mimics (GenePharma, Shanghai, China) were added dropwise, along with an equal volume of vacant cationic liposome suspension and incubated for 0.5 h with gentle shaking at 37 °C.

### 2.15. Statistical Analysis

All of the data are presented as the mean ± SEM with GraphPad Prism 6 (GraphPad Software Inc., La Jolla, CA, USA). Every experiment was independently conducted at least three times. Differences were regarded as statistically significant at *p* < 0.05 (*) and very significant at *p* < 0.01 (**). Student’s *t*-test was used to compare the data from pairs of treated and untreated groups.

## 3. Results

### 3.1. Exosomes Isolated from NDV-Infected HeLa Cells Cause a Cytopathic Effect

**Figure 1:**
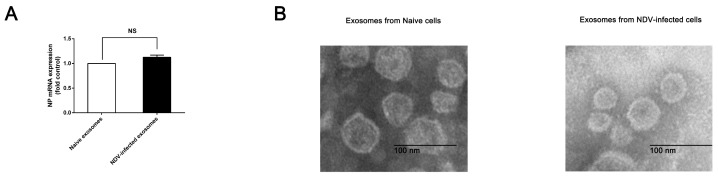
(**A**) Exosomes were extracted from naive and Newcastle disease virus (NDV)-infected HeLa cells. (**B**) The images of exosomes secreted by NDV-infected and naive HeLa cells were obtained using TEM. The size bar is 100 nm.

In order to investigate the role of exosomes in NDV infection, we extracted exosomes from naive cells and NDV-infected HeLa cell culture supernatants based on differential ultracentrifugation. Distinguishing between viruses and exosomes has always been difficult. NDV has a characteristic of agglutinating red blood cells. According to this characteristic, we used chicken red blood cells to adsorb NDV from exosome samples. A hemadsorption test (HA) was used to detect the effectiveness of this adsorption method. We then used RT-qPCR to detect NDV nucleocapsid protein (NP) mRNA in these exosome samples. The results showed that the expression of NP in the exosomes from NDV-infected cells was not significantly different from that of uninfected cells, which suggested that NDV was successfully removed from the exosome samples (Figure 1A). Any cells or debris left in the samples were removed with a 0.22 μm polyvinylidene difluoride (PVDF) membrane. The morphology of these particles was observed by TEM. We found that the particles had the lipid bilayer, and were round in shape, and approximately 120 nm in size (Figure 1B).

**Figure 2:**
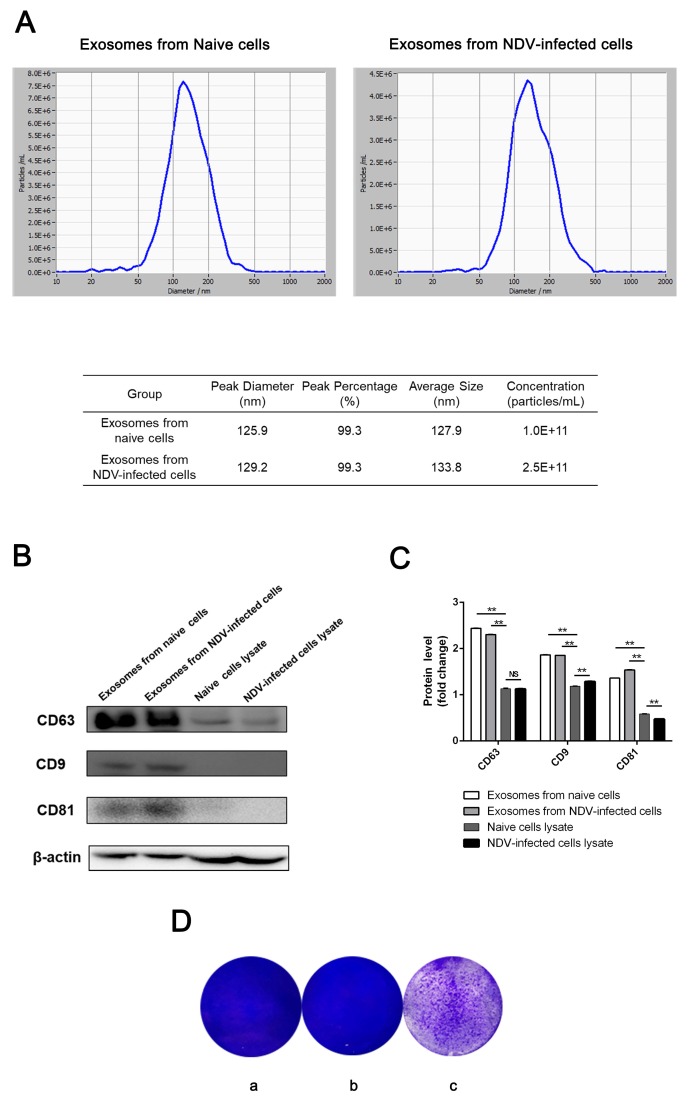
Characterization of exosomes were identified from NDV-infected and naive HeLa cells. (**A**) Size and concentration of exosomes from NDV-infected and naive HeLa cells were measured using nanoparticle tracking analysis. (**B**) CD63, CD9, and CD81 proteins in the exosomes derived from NDV-infected and naive HeLa cells were detected by Western blotting. The expression of these proteins in naive HeLa cell lysates was used as the control. (**C**) Relative expression of the CD63, CD9, and CD81 proteins from (C) was analyzed with Image J software 1.8.0. β-actin was used as an internal control. (**D**). (**a**) Image of plaques formed in HeLa cells infected with NDV alone, (**b**) treated with exosomes derived from naive cells and then infected with NDV, and (**c**) treated with exosomes derived from NDV-infected cells and then infected with NDV.

The results of the NTA indicated that the median size of these particles from naive cells was 127.9 nm, and the percentage of particles that were 125.9 nm in size was 99.3%. The median size of the particles from the NDV-infected HeLa cells was 133.8 nm, and the percentage of particles that were 129.2 nm in size was also 99.3% (Figure 2A). Our NTA results were consistent with the particle sizes observed by TEM. Additionally, the concentrations of exosomes derived from NDV-infected and naive HeLa cells were 2.5 × 10^11^ particles/mL and 1.0 × 10^11^ particles/mL, respectively. The former concentration was approximately 2.5 times the latter one, which may have resulted from the effect of NDV stimulation on exosome secretion. CD63, CD9, and CD81, exosomal marker proteins, were abundant in HeLa exosome samples, while their expression in HeLa cell lysates significantly decreased (Figure 2B,C). These results confirm that the pellets from NDV-infected and naive HeLa cells were exosomes, and thus, these samples were prepared for further study. HeLa cells were incubated with these particles for 24 h and then infected with NDV to observe the cytopathic effects. To our surprise, the cytopathic effect was significantly observed in cells treated with exosomes from NDV-infected cells (Figure 2D).

### 3.2. NDV Infection Affects Exosomal miRNA Expression

**Figure 3:**
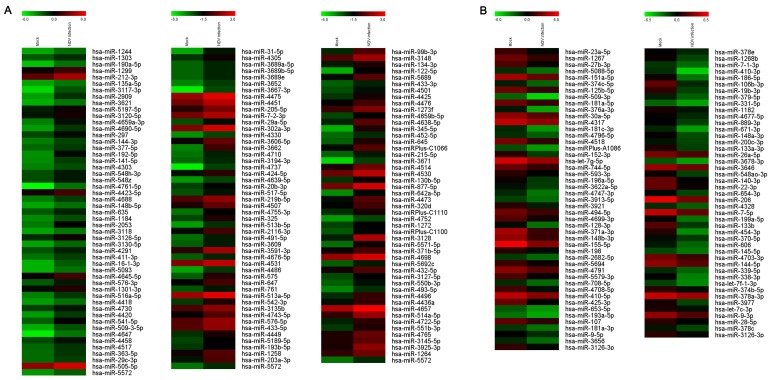
NDV infection alters host miRNAs expression in exosomes derived from HeLa cells. (**A**) Heat maps show miRNA expression that was upregulated more than 1.5-fold in exosomes derived from HeLa cells 24 h after NDV infection. (**B**) Heat maps show miRNA expression that was downregulated more than 1.5-fold in exosomes derived from HeLa cells 24 h after NDV infection.

To profile the role of exosomal miRNA in NDV infection, total RNA from uninfected and NDV-infected HeLa cell-derived exosomes was analyzed using microarrays. It was observed that the expression of 234 host miRNAs changed more than 1.5 times (Figure 3A), and 144 of them were upregulated (Figure 3B). The microarray results suggested that NDV infection induced ectopic expression of a large number of host miRNAs in exosomes.

### 3.3. MicroRNAs Affect the Expression of IFN-β and Interferon Stimulated Genes (ISGs)

**Figure 4:**
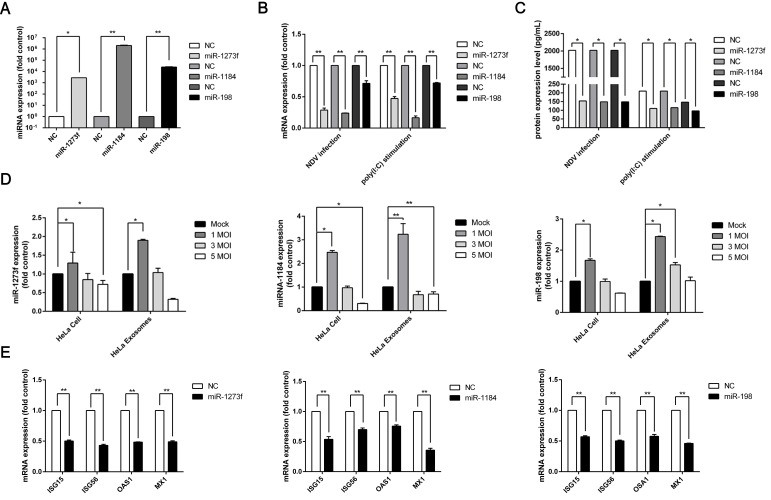
MicroRNAs affect interferon (IFN)-β and interferon stimulated gene (ISG) expression. (**A**) The expression level of three miRNAs in HeLa cells transfected with miRNA mimics was detected by RT-qPCR. (**B**) Three miRNAs (miR-1273f, miR-1184, and miR-198) or negative control miRNA (NC) were transfected into HeLa cells. The HeLa cells were then infected with NDV at a multiplicity of infection (MOI) of 1 or treated with 50 μg/mL poly (I:C). The IFN-β mRNA level was detected with RT-qPCR. (**C**) Three miRNAs (miR-1273f, miR-1184, and miR-198) or NC were transfected into HeLa cells, and then these cells were infected with NDV at a MOI of 1 or treated with 50 μg/mL poly (I:C). The IFN-β protein expression was detected by enzyme-linked immunosorbent assay (ELISA). (**D**) The expression of three miRNAs in HeLa cells infected with NDV and exosomes derived from NDV-infected and naive HeLa cells was detected by RT-qPCR. (**E**) Three miRNAs (miR-1273f, miR-1184, and miR-198) or NC were transfected into HeLa cells, and then these cells were infected with NDV at a MOI of 1. The ISG (*ISG15, ISG56, OSA1,* and *Mx1*) expression was detected by RT-qPCR. Error bars are the SEM from three independent experiments. Statistical significance is indicated: ** *p* <  0.01 and * *p* <  0.05. ISGs are usually transcribed by the activation of the Janus kinase (JAK)-signal transducer and transcription (STAT) pathway with canonical type I IFN signaling activities [30]. While IFN-β expression was affected by the three miRNAs, the downstream ISG expression may also be affected. To prove our hypothesis, the detection of ISG expression in HeLa cells overexpressing these three miRNAs was analyzed by RT-qPCR after infection with NDV. The results indicated that the mRNA level of ISGs, such as *ISG15*, *ISG56*, *OSA1*, and *Mx1*, was significantly downregulated in the cells treated with the miRNA mimics (miR-1273f, miR-1184, and miR-198) during NDV infection compared to cells treated with NC miRNA (Figure 4E). It demonstrated that these three miRNAs (miR-1273f, miR-1184, and miR-198) might affect the expression of IFN-β as well as the expression of ISGs.

IFN-β production is an inaugural event that occurs in host innate immune responses during viral infection. Only a minimal change in IFN-β expression is needed to activate crucial antiviral signaling cascades [22]. However, NDV replication still increases rapidly instead of being substantially inhibited, despite the abundant production of IFN-β during NDV infection. The expression of many microRNAs has been altered due to NDV infection, and whether or not some microRNAs have an effect on IFN-β expression is not known. To explore this issue, miRNAs were chosen for further study based on the results that they were predicted to target IFN-β by TargetScan and miRDB. HeLa cells were transfected with these miRNA mimics and then treated with poly (I:C) for 24 h, which is an IFN-β inducer. Then the IFN-β mRNA level in the HeLa cells and the IFN-β protein level in the cell supernatants were tested by RT-qPCR and an ELISA, respectively. The results indicated that during NDV infection in HeLa cells overexpressing three of these miRNAs—miR-198, miR-1184, and miR-1273f—the IFN-β mRNA and protein levels decreased significantly compared with those in the HeLa cells overexpressing negative control (NC) RNA (Figure 4A–C). 

To further explore the effect of these three miRNAs on IFN-β expression during NDV infection, we replaced poly (I:C) stimulation with NDV infection. We obtained similar results, such that overexpression of the three miRNAs affected IFN-β expression (Figure 4B,C). To verify the data analyzed by the microarray, the expression of the three miRNAs in HeLa cells and exosomes secreted by those cells was detected with RT-qPCR. The results obtained by RT-qPCR were consistent with the microarray findings (Figure 4D), indicating that NDV infection could induce abnormal expression of exosomal miRNA. The expression of miRNAs manifested a trend towards downregulation with an increase in NDV infection, and we conjectured that massive cell death hinders some responses of these cells to NDV infection in a time-dependent manner, such as altering the expression of host microRNAs, with the increasing MOI of NDV. 

### 3.4. MicroRNAs May Target IFN-β

**Figure 5:**
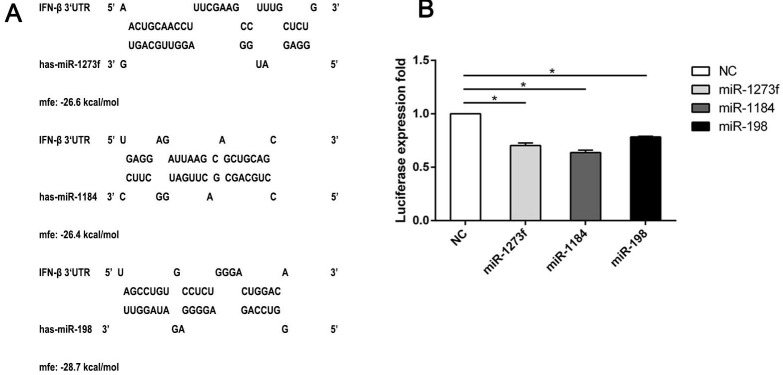
miRNAs (miR-1273f, miR-1184, and miR-198) may interact with the predicted target sites in the 3′ UTR of IFN-β mRNA. (**A**) Predicted target sites for miR-1273f, miR-1184, and miR-198 in IFN-β 3′ UTR mRNA were shown. Nucleotide positions in the 3′ UTR, which were complementary to the miR-1273f, miR-1184, and miR-198 seed region, were indicated. (**B**) Effect of miR-1273f, miR-1184, and miR-198 mimics on the luciferase expression of reporter constructs containing the putative miRNA binding sites from the IFN-β 3′ UTR was shown. The values were expressed as the mean of the results from three independent experiments, with the error bar showing standard deviations. Statistical significance is indicated: * *p* <  0.05.

These three selected miRNAs not only affected the transcription and translation of IFN-β, but they also affected the transcription of downstream effectors, including ISGs. However, the mechanism behind the phenomena remains unknown. Furthermore, it was unknown if the miRNAs influenced directly or indirectly the expression of IFN-β and ISGs. Interaction sites with the miRNA “seed region” are primarily within the 3′ UTR of a gene mRNA and rarely within their 5′ UTR [31]. Therefore, we focused on the interaction between the 3′ UTR of IFN-β and the three selected miRNAs. First, we used bioinformatics software to predict the interaction sites between these three miRNAs and IFN-β 3′ UTR. We found that the three miRNAs could directly interact with the IFN-β 3′ UTR (Figure 5A). To further verify the relationship between IFN-β and the miRNAs, we cloned the IFN-β 3′ UTR sequence containing the predicted miRNA interaction sites into a dual luciferase expression vector and co-transfected it with miRNA mimics into human 293T cells. In 293T cells, the three candidate miRNAs (miR-1273f, miR-1184, and miR-198) reduced significantly the luciferase expression compared to the negative control (Figure 5B). In conclusion, the three miRNAs we selected directly interacted with the 3′ UTR of IFN-β. This result also proved indirectly that the downregulation of ISG expression is probably due to targeting IFN-β 3′ UTR by the three miRNAs. The direct interactions between the ISG 3′ UTR and these three miRNAs remain to be elucidated.

### 3.5. Exosomes Could Carry MicroRNAs to Promote NDV Diffusive Replication

**Figure 6:**
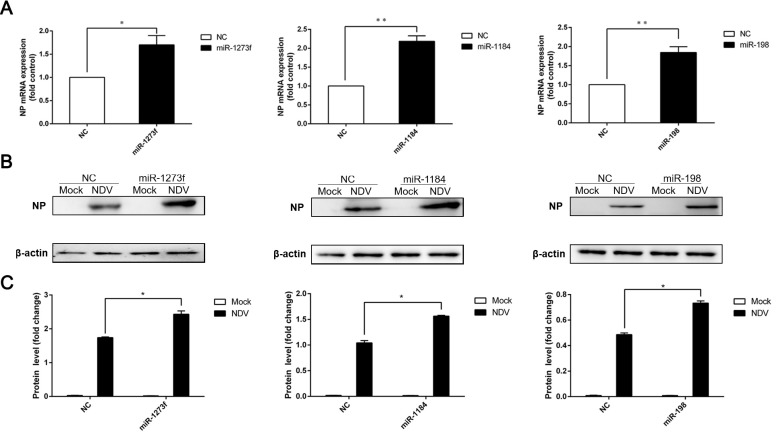
MicroRNAs benefit NDV replication. (**A**) Nucleocapsid protein (NP) mRNA was detected with RT-qPCR in HeLa cells transfected with miRNA mimics and then infected with NDV. β-Actin was an internal control. (**B**) NP protein was detected by Western blot in HeLa cells transfected with miRNA mimics and then infected with NDV. β-actin was an internal control. (**C**) The integrated density of NP expression was analyzed in (**B**) with Image J software 1.8.0. β-actin was an internal control. Error bars are the SEM from three independent experiments. Statistical significance is indicated: ** *p* <  0.01 and * *p* <  0.05.

The above results indicated that the three miRNAs (miR-1273f, miR-1184, and miR-198) we selected might affect the expression of IFN-β. In previous studies, it was found that some host miRNAs were able to affect viral replication. For instance, miR-122 promoted HCV replication [32]. To explore whether these three miRNAs (miR-1273f, miR-1184, and miR-198) were able to affect the replication of NDV, HeLa cells were transfected with the miRNA mimics at first, and then the cells were infected with NDV at a MOI of 1. We detected the mRNA and protein levels of NP gene by RT-qPCR and Western blot, respectively. RT-qPCR results demonstrated that all three miRNAs increased significantly the level of NP mRNA (Figure 6A). In accordance with the above results detected by RT-qPCR, these three miRNAs were also demonstrated to increased NDV NP protein by Western blot (Figure 6B,C). Therefore, miR-1273f, miR-1184, and miR-198 were able to affect NDV replication.

The above results reflect how abnormal miRNA expression in HeLa cells affects NDV replication. However, it is still unknown if miRNAs are beneficial for not only the spread of NDV into the extracellular space, but also for the intracellular infection of NDV. It has been reported that in some viral infections, exosomes hijacked by viruses transmitted miRNAs promoting viral replication, which was conducive to the spread of the virus [33]. We put forward a hypothesis that miRNAs may be transferred into bystander cells via exosomes, and thus, play a potential role in NDV infection.

**Figure 7:**
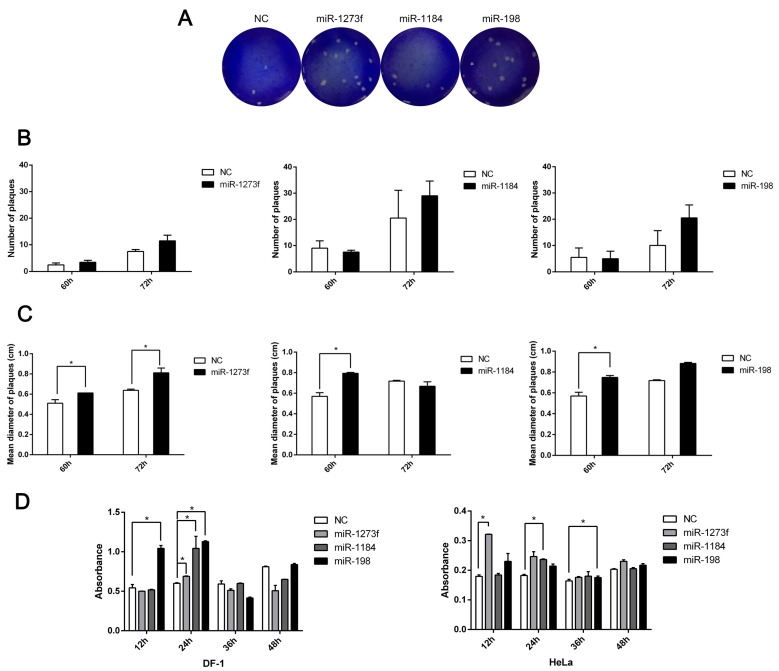
Exosomes carrying miRNAs promote NDV spread. (**A**) NDV titers were detected by plaque assay in DF-1 cells transfected with miRNA mimics and then infected with NDV. (**B**) The average number of plaques was calculated in DF-1 cells transfected with miRNA mimics and then infected with NDV by the Image-Pro Plus 6.0 software. (**C**) The mean diameter of plaques in (**B**) was measured in DF-1 cells transfected with miRNA mimics and then infected with NDV by the Image-Pro Plus 6.0 software. (**D**) Absorbance was detected by ELISA in DF-1 cells and HeLa cells were transfected with miRNA mimics and then infected with NDV. Error bars are the SEM from three independent experiments. Statistical significance is indicated: ** *p* <  0.01 and * *p* <  0.05.

To prove this hypothesis, we attempted to simulate the process whereby miRNA-loaded exosomes transfer these miRNAs between different cells during NDV infection. However, at the time of our study, there were no exosome products consisting of coated miRNAs commercially available. Therefore, we prepared miRNA-loaded liposomes that were used to simulate this process, as there are several similarities between liposomes and exosomes, including fusion with the cell membrane and delivery of RNAs. The average size of the liposomes prepared by dynamic light scattering was approximately 100 nm, similar to that of the exosomes. Since microRNAs usually regulate target genes in a delicate manner, and the NDV plaques in HeLa cells were difficult to count, DF-1 cells were used for observing the NDV plaques. After incubating for 24 h with miRNA-loaded liposomes, DF-1 cells were infected with diluted NDV. Then we selected the optimal viral dilution for forming plaque units. Six different infection times (12, 24, 36, 48, 60, and 72 h) were selected to observe the size and number of plaque-forming units based on the most appropriate virus dilution. We found that plaques on DF-1 cells appeared after 60 h and no plaques formed with NDV infection for 12–48 h (Figure 7A). After 72 h of infection, the number of the plaques on the DF-1 cells slightly increased (Figure 7B). Of these plaques, the diameters on DF-1 cells incubated with miRNA-1273f increased significantly at 60 and 72 h (Figure 7C). The significantly increased plaque diameter was only observed on DF-1 cells incubated with miRNA-1184 and infected with NDV for 60 h and DF-1 cells incubated with miRNA-198 and infected with NDV for 72 h (Figure 7C). DF-1 cells incubated with NC-loaded liposomes were used as a control. These findings suggested that NDV may utilize exosomes to transport miRNAs into neighboring cells, making the cells more susceptible to NDV infection, but the effect was very subtle and not obvious. Furthermore the exact effect remains to be elucidated. In order to detect the effect of these microRNAs in enhancing NDV replication on HeLa cells, HeLa cells were cultured in 96 cell culture plates and transfected with microRNA mimics (miR-1273f, miR-1184 and miR-198). After 24 h, these cells were infected with Herts/33 (a strain of NDV). These supernatants were removed at 12, 24, 36, and 48 h after NDV infection, respectively and fixed with 4% formaldehyde. Finally, the absorption of these cells was tested at 450 nm with ELISA. The results are shown in Figure 7D. From Figure 7D, the effect of microRNA in enhancing NDV replication on HeLa cells really exists despite the effect which is not stronger than on DF-1 cells. The reduced effect from DF-1 cells to HeLa cells may be due to the difference in susceptibility of NDV infection.

## 4. Discussion

Exosomes are secreted by almost all cells, with their contents comprising of nucleic acids, proteins, and lipids [34]. It has been shown that the packaging of these contents into exosomes has preference and specificity, not randomness [35]. In our study, microarray analysis demonstrated significant regulation of the expression of 234 miRNAs in exosomes secreted from NDV-infected HeLa cells compared with that from naive cells. Furthermore, miR-1273f, miR-1184, and miR-198 played a vital role in disseminating NDV infection to neighboring cells by targeting directly the 3′ UTR region of IFN-β. 

We found that the classic exosome isolation method, according to Thery et al. [24] with minor modifications on centrifugal velocity, harvested the most exosomes. In order to exclude the NDV particles from exosomes, we exploited the HA test to purify the exosomes. The morphology of the purified exosomes was identified based on TEM and their size and concentration were analyzed with NTA. In order to eliminate the effect of other ingredients in exosomes on this process, we had to design a type of artificial exosome with the guidance of the literature [36]. These artificial exosomes were about 100–150 nm in size and had lipid bilayer membranes, which are similar to natural exosomes. Since these artificial exosomes lack specific targeting signals to recipient cells and were devoid of other cellular material (aside from the miRNAs), we used these exosomes to study the effect of specific miRNAs on NDV infection. Natural exosomes exhibit less harm to recipient cells, but the purification of natural exosomes requires a large number of samples and high-level isolation technology. Moreover, other molecules in natural exosomes could interfere with our study regarding these specific microRNAs. The microRNAs of interest were packaged by artificial exosomes and showed a positive effect on NDV replication (Figure 7); however, their combined effect on NDV infection is still not known.

**Figure 8:**
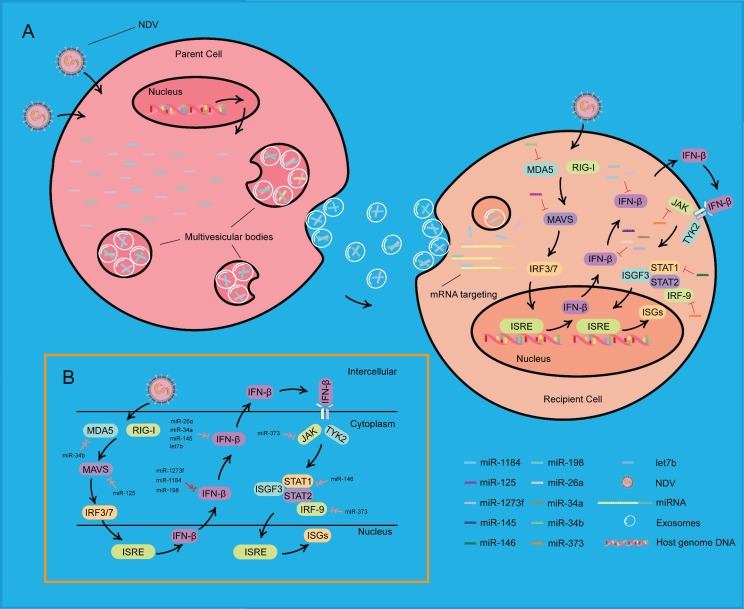
Schematic showing that exosomes carrying miRNAs promote NDV infection. (**A**) During NDV infection, three miRNAs changed their expression and loaded into exosomes derived from multivesicular bodies (MVBs) of a NDV-infected HeLa cell, called the “parent cell”. MVBs fuse with the plasma membrane and release exosomes that contain these miRNAs. In another HeLa cell, called the “recipient cell”, these three miRNAs, transferred by exosomes, affect the IFN-β mRNA and further affect the expression of ISGs, eventually resulting in increasing NDV replication. (**B**) MicroRNAs may be involved in the IFN I pathway during NDV infection. MicroRNAs marked by the red arrow were found in the current research, and microRNAs marked by the pink arrow were found in other virus infections and remain to be elucidated.

As shown in Figure 8, when NDV invades a HeLa cell, the nucleus of this cell receives a signal from the cytoplasm, which results in a change in microRNA expression. These microRNAs are loaded into exosomes and secreted by the cell, called the “parent cell”. These exosomes then enter into another HeLa cell, called the “recipient cell”. These microRNAs are released into the cytoplasm of the recipient cell. Once NDV invades the recipient cell, the viral RNA is recognized specifically by retinoic acid-inducible gene I (RIG-I) and melanoma differential gene 5 (MDA5). These microRNAs, transferred by exosomes, bind to the 3′ UTR of IFN-β and inhibit the antiviral effect in the cytoplasm of the recipient cell. Thus, the recipient cell exhibits increased susceptibility to NDV infection.

NDV is also an oncolytic virus [37]. NDV infection usually has minimal clinical symptoms, which makes this virus an optimal virus in the treatment of human cancers [38,39]. When NDV invades normal human cells, it elicits a rapid and strong type I IFN response [40]. This response protects cells from viral replication and cytotoxic effects of NDV, which is the reason for the high safety rating in clinical applications in humans [41]. However, human cancer cells are relatively permissive to the infection of NDV, and the continued replication of this virus has great potential to kill cancer cells. Studies have shown that NDV used its full-length anti-genome as a template or inhibited RIG-I pathway to support replication [42]. In this current study, exosomes from neighboring cells also promoted NDV replication. During NDV infection, the expression of microRNAs changed. The microRNAs were then loaded into exosomes and released into other cells to inhibit IFN-β signaling, forming a micro-environment that was susceptible to NDV infection, thereby contributing to cancer apoptosis.

In previous studies, many miRNAs were reported to affect viral infection. For example, miR-198 was reported to repress HIV infection by targeting cyclin D1 [43], which regulates cell cycle transition [44]. MiR-146 was transferred by exosomes to target IRAK1, TRAF6, and STAT1, and suppress IFN production in target cells to facilitate EV71 infection [33]. In addition, miRNAs have been demonstrated to be involved in other avian diseases, such as avian leukosis virus subgroup J (ALV-J). Li et al. found that miR-23b could target IRF1 and promote ALV-J replication [45]. Liu et al. demonstrated that chicken IRF1 activates IFN-β to suppress NDV replication, and it was involved in chicken antiviral innate immunity [46]. Thus, it could be speculated that miR-23b might suppress IFN-β expression through chicken IRF1, thereby supporting NDV replication. In other studies, microRNAs were shown to have negative roles in the IFN response during other viral infections (Figure 8). Moreover, their roles in NDV replication remains to elucidated. In the current research, we found a role of miR-198, miR-1273f, and miR-1184 in targeting IFN-β during NDV infection.

Taken together, we suggest that exosomes play a positive role in viral infection. To our knowledge, this is the first report regarding the involvement of exosomes from the oncolytic NDV in tumor cells. The results from this study provide new insights for the development of anti-tumor therapies.

## Figures and Tables

**Table 1:** Sequence of gene mRNA primers for RT-qPCR.

Gene Symbol	GenBank Accession No.	Forward Primer Sequence (5′ to 3′)	Reverse Primer Sequence (5′ to 3′)
***IFN-β***	NM_002176.3	CATTACCTGAAGGCCAAGGA	CAATTGTCCAGTCCCAGAGG
***ISG15***	NM_005101	AATGCGACGAACCTCTGAAC	GAAGGTCAGCCAGAACAGGT
***ISG56***	NM_001548	GCAGCCAAGTTTTACCGAAG	AGCCCTATCTGGTGATGCAG
***OSA1***	AF521670.1	CCAGGAAATTAGGAGACAGC	GAGCGAACTCAGTACGAAGC
***Mx1***	NM_001178046.2	GGTGGTGGTCCCCAGTAATG	ACCACGTCCACAACCTTGTCT
***NDV NP***	ARJ54653.1	CGGTATTCACTCTTAACAATG	CCTCACTAACAGCAATCC
***β-actin***	HQ154074	GGCATCCTCACCCTGAAGTA	AGGTGTGGTGCCAGATTTTC

**Table 2:** Specific miRNA primers for RT-qPCR.

miRNA Name	miRNA Sequence ^a^ (5′-3′)	miRbase Accession No.	RT Primer Sequence (5′-3′)	Forward PCR Primer Sequence (5′-3′)
**hsa-miR-1273f**	GGAGAUGGAGGUUGCAGUG	MIMAT0020601	GTCGTATCCAGTGCAGGGTCCGAGGTATTCGCACTGGATACGACCACTGC	CGCGCGGGAGATGGAGGTT
**hsa-miR-1184**	CCUGCAGCGACUUGAUGGCUUCC	MIMAT0005829	GTCGTATCCAGTGCAGGGTCCGAGGTATTCGCACTGGATACGACGGAAGC	GCGCCTGCAGCGACTTGATG
**hsa-miR-198**	GGUCCAGAGGGGAGAUAGGUUC	MIMAT0000228	GTCGTATCCAGTGCAGGGTCCGAGGTATTCGCACTGGATACGACGAACCT	TGCGGGTCCAGAGGGGAGAT

^a^ The reverse PCR primer of various miRNAs is universal, and the sequence is 5′-ATCCAGTGCAGGGTCCGAGG-3′.
